# Efficacy and Safety of Direct-Acting Oral Anticoagulants (DOACs) in the Overweight and Obese

**DOI:** 10.1155/2020/3890706

**Published:** 2020-05-23

**Authors:** Kimberley Doucette, Hira Latif, Anusha Vakiti, Eshetu Tefera, Bhavisha Patel, Kelly Fitzpatrick

**Affiliations:** ^1^Medstar Washington Hospital Center, Departments of Internal Medicine and Hematology and Oncology, Washington, DC, USA; ^2^Medstar Georgetown University Hospital, Departments of Hematology and Oncology, Washington, DC, USA; ^3^Medical College of Georgia, Department of Hematology and Oncology, Augusta, GA, USA; ^4^Medstar Health Research Institute, Biostatistics and Biomedical Informatics, Hyattsville, MD, USA; ^5^National Heart, Lung and Blood Institute, Hematology Branch, Bethesda, MD, USA

## Abstract

Obesity plays an essential role in the safety of pharmacologic drugs. There is paucity of data for direct oral anticoagulants (DOACs) in the obese, despite these agents becoming more widely used. The primary and secondary objectives of this study were to assess the safety and efficacy of DOACs in the overweight and obese populations when used for primary prophylaxis in the setting of non-valvular atrial fibrillation (NVAF) and for treatment of venous thromboembolisms (VTE). We conducted a retrospective cohort study in a large tertiary care center and obtained data through review of electronic health records. Among patients with NVAF and VTE on apixaban, there were no differences in rates of major bleeding (MB) and clinically relevant nonmajor bleeding (CRNMB) in the overweight and obese populations when compared to normal weight and underweight individuals. The multivariate adjusted analysis for rivaroxaban found that the odds of CRNMB for patients with BMI <25 was 5.37 (95% CI 1.50–19.32) times higher than that of BMI ≥25. Moreover, patients on medications that had known interactions with DOACs had 6.40 times higher odds of CRNMB than patients without such interactions (95% CI 1.49–27.57), which was not accounted for by the effects of aspirin and plavix alone. Efficacy was similar between all weight groups, for both apixaban and rivaroxaban. These results support previous analyses preformed in the large phase III trials and confirm that apixaban and rivaroxaban are safe in the overweight and obese.

## 1. Introduction

Obesity is a major healthcare challenge that is estimated to escalate to a global epidemic in the following years [1]. Therefore, it is essential to assess the healthcare needs of this population in terms of pharmacologic modifications required for this subset to provide effective treatment and prevent adverse events. Obesity is a well-established risk factor for developing VTE (venous thromboembolism), and vitamin K antagonists (VKA) have historically been the mainstay of anticoagulation treatment [2–5] given lack of data in this specific population. Direct oral anticoagulants (DOAC) are now approved for use as primary prophylaxis in patients with nonvalvular atrial fibrillation to prevent stroke and systemic embolism, as well as treatment for deep vein thrombosis (DVT) and pulmonary embolism (PE). They are also deemed safe for use as prophylaxis for venous thromboembolism (VTE) in patients undergoing knee or hip replacement surgery. These newer agents have shown equal efficacy as low-molecular weight heparin (LMWH) and VKAs [6, 7]. In addition, studies have shown no differences in efficacy to VKAs or LMWH in overweight and obese patients [8–13]. Additionally, safety parameters, namely, major bleeding and clinically relevant nonmajor bleeding (CRNMB), are reported to be lower in all patients taking DOACs when compared to VKAs [14]. Their fixed dosing, limited dietary interactions, and reduced need for routine monitoring make them a convenient alternative to the traditional VKA.

Subgroup analyses in various randomized clinical trials have evaluated the efficacy and safety measures in obese patients and showed no differences in efficacy compared to conventional VKAs or enoxaparin. Fourteen percent of the patients in the EINSTEIN trial and 20% in the AMPLIFY trial were over 100 kg, and the results for efficacy and safety outcomes were consistent and did not suggest a need for dose modification for weight. However, there have been no dedicated large prospective randomized control trials to address the use of DOAC in the obese. Despite limited data, these agents are being used in clinical practice in the real world population [8–13, 15–17].

Pharmacokinetics and pharmacodynamics studies in this domain have been conflicting and not conclusive. In a healthy volunteer study, an inverse relationship between the drug and patient weight was observed [9, 18]. Drug exposures were found to be decreased along with reduced concentrations and shortened half-lives (time to steady state) in patients with BMI >40 kg/m^2^. Other smaller studies and case reports have demonstrated stable pharmacokinetics and pharmacodynamics in obese patients for both rivaroxaban and apixaban [7, 19, 20]. To date, there exists no strong evidence of clinical efficacy and safety in obese patients on therapeutic DOACs. As such, the general recommendations are to avoid DOACs in the extremes of weight (e.g., <50 kg, >120 kg, or BMI ≥35 kg/m^2^) unless VKA is contraindicated [21–23]. In addition, there are some concerns about their interaction with other cytochrome p-450 3A4 modifying medications such as amiodarone, verapamil, antifungal agents such as ketoconazole, and antibiotics such as erythromycin in obese patients [24].

Given the lack of robust clinical evidence of using these newer agents in the obese population, we aim to evaluate the efficacy and safety of DOACs (apixaban, rivaroxaban, and dabigatran) in overweight and obese patients with NVAF and VTE. Our study was performed in the diverse population of patients being treated at the largest hospital in the District of Columbia.

## 2. Methods

We conducted a retrospective cohort study of the inpatient and outpatient population at Medstar Washington Hospital Center in Washington DC, recruited between January 2014 and December 2015. Patients were included if prescribed DOACs (either rivaroxaban, apixaban, or dabigatran) for VTE, NVAF, or stroke prophylaxis and were 18 years or older. Exclusion criteria were inconsistent follow-up or documented noncompliance after initiation of therapy. We collected baseline demographic data including age, race, gender, BMI, length of follow-up time, and comorbidities. We also assessed concomitant use of CYP3A inhibitors and inducers (nondihydropyridine calcium channel blockers, amiodarone, antiarrhythmics, protease inhibitors, and antiepileptic (carbamazepine/phenytoin)) and antiplatelets (aspirin and/or clopidogrel).

Our primary endpoint was safety events up to 2 years after initiation of DOAC for NVAF patients and 6 months for VTE patients in overweight and obese patients (BMI <25 versus ≥25 and <30 versus ≥30, respectively). Safety measures were defined as any bleeding defined by the International Society on Thrombosis and Haemostasis (ISTH) criteria. Major bleeding was defined as overt bleeding with a drop in hemoglobin of at least 2 g/dL or requiring 2 units of packed red blood cells at a critical site. Clinically relevant nonmajor bleeding (CRNMB) was defined as clinically overt bleeding leading to hospitalization, medical/surgical intervention, or change in anticoagulation treatment but did not satisfy criteria of major bleeding [25]. Combined bleeding included both CRNMB and MB. Our secondary outcome was efficacy and included strokes for the NVAF cohort and recurrent VTE in the VTE cohort with follow-up up to 2 years and 6 months, respectively, after initiation of a DOAC. Patients were stratified by BMI (kg/m^2^) to assess the efficacy and safety in the obese patient population. BMI categories include normal range (18–24.99), overweight (25–29.99), obese class I (30–34.99), obese class II (35–39.99), and obese class III (>40.00) per WHO criteria.

Summary statistics were performed on baseline characteristics in the various study groups using means, standard deviations, and proportions (if categorical). A two sample *t*-test was used to examine differences in the averages of continuous variables between groups (NVAF vs VTE). Chi-square and Fisher's exact tests were used as appropriate to investigate differences for categorical variables.

To assess the relations between outcomes and BMI, our statistical analyses included chi-square, Fisher's exact (when cells had fewer than 5 counts), and logistic regression. If patients had missing variables, they were removed from analyses. We also performed analysis on combined BMI groups, <25 versus ≥25 and <30 versus ≥30. Our logistic regression models adjusted for the comorbidities, dose of DOAC, and use of CYP3A inhibitors and inducers or antiplatelet agents. A *p* value <0.05 was considered statistically significant. We used the SAS statistical software.

## 3. Results

### 3.1. Patient Characteristics

We collected data for a total of 398 patients from January 2014 to December 2015 who received DOACs for NVAF or VTE at Medstar Washington Hospital Center (MWHC) in either an inpatient or outpatient encounter. The patient demographics are described in Table 1. In the NVAF cohort, males were slightly overrepresented (57%) and median age was 72 years. Seventy-nine percent of the patients had BMI >25 and 47% had BMI >30, with 13% having a BMI >40.

In contrast, the VTE cohort had slight predominance of females (58%) with a median age of 65 years. African Americans were majority, with a similarly large portion of patients having BMI >25 (75%), and 26% with BMI >30, and 12% with a BMI >40. The two groups differed with respects to history of hypertension, heart failure, and presence of interacting medications and use of aspirin or clopidogrel; the NVAF group had a larger proportions of all these factors. Rivaroxaban was prescribed more commonly in the VTE group (79%) compared to the NVAF group (44%). Only a small fraction of patients were on dabigatran in both groups.

### 3.2. NVAF Primary Outcome: Safety

When BMI <30 was compared to BMI ≥30, in the apixaban and rivaroxaban groups, rates of bleeding (major, CRNMB, and combined bleeds) were not statistically different. In contrast, CRNMB and combined bleeds (CRNMB and MB) in the rivaroxaban group were more frequent in patients with normal BMI (<25) compared to overweight BMI (>25) (*p* = 0.0004) (Table 2). A logistic regression model was then conducted adjusting for age, sex, race, prior stroke, presence of interacting medications, dose of DOAC, and history of prior bleeds. This multivariate analysis showed that when adjusting for these covariates, the odds of CRNMB for patients with BMI <25 was 5.37 (95% CI 1.50–19.32) times higher than that of patients with BMI ≥25 (Table 3). It also showed that patients with drug interactions had 6.4 times higher odds of CRNMB than patients without the presence of drug interactions (95% CI 1.49–27.57). Neither aspirin nor clopidogrel alone accounted for this effect. The same multivariate analysis for patients with BMI <30 versus ≥30 showed no statistically significant difference in rates of CRNMB (Table 4). For apixaban, comparing BMI <25 to BMI ≥25, both CRNMB and MB rates were found to be similar between the groups. The sample size for dabigatran was too small, and therefore, no statistical analyses could be performed on these patients.

### 3.3. VTE Primary Outcome: Safety

For rivaroxaban in the VTE cohort, bleeding rates were similar across all BMIs. There was no significant relationship across the different BMI groups for major bleeding, CRNMB, and combined bleeding (Table 5). The sample sizes for these groups were smaller than the NVAF cohort. The sample size for dabigatran and apixaban were too small, and therefore no statistical analysis could be performed on these patients.

### 3.4. Secondary Outcomes: Efficacy

For both apixaban and rivaroxaban in the NVAF cohort, stroke rates were similar across all BMIs; however, TIA events were uncommon in the rivaroxaban group, making an analysis impossible. Fisher's exact test revealed no significant relationship across the different BMI groups and stroke/TIA rates (Table 6). For the VTE cohort, recurrence rate of VTE at 6 months appeared similar across all BMIs for both apixaban and rivaroxaban. Sample size was again very small for the apixaban group (19 patients in total). Fisher's exact test for recurrence rate in the apixaban group and chi-square for recurrence rate in the rivaroxaban group revealed no significant relationship across the different BMI groups (Table 6).

## 4. Discussion

Due to paucity of data regarding use of DOACs in the obese population, we have to base our clinical decisions about their use on available evidence. Our study provides further evidence of comparable efficacy and safety of apixaban and rivaroxaban in the obese patients with AF and VTE. Our results suggest that there is an increased rate of clinically relevant nonmajor bleeding in patients on rivaroxaban with normal or lower weights (BMI <25) as opposed to overweight and obese patients, independent of dose of DOAC. This finding was not seen in the apixaban group. We found no increased bleeding risk in obese patients with BMI ≥30 compared to patients with BMI <30. Our sample size was too small to assess the safety profile of patients with a BMI over 40; however, we did include a high proportion of overweight and obese patients. Our limitations are that it is a retrospective study with potential for selection bias and has a small sample size. We were unable to collect enough data for dabigatran in the VTE and AF cohorts or for apixaban in the VTE cohort. To fully assess efficacy, a longer follow-up period will be required. Our safety results support the current literature and represent a generalizable tertiary center population. Unfortunately, a lot of bleeding episodes occurred in outpatient setting, and we were unable to collect data on coagulation studies. Another limitation is the lack of documentation on herbal supplements taken over the counter, which may have interactions with DOACs. We collected data on use of over the counter aspirin, which is often documented in progress notes, though is not linked with pharmacy records, making documentation variable. A recent large retrospective study, looking at safety measures and efficacy in the extreme obesity weight class (morbidly obese) on DOACs, found no increased bleeding risk and similar efficacy compared to patients on warfarin [26]. There was another retrospective study in 2016, that showed no significant difference between rates of bleeding when comparing patients that were underweight (<60 kg) versus those with a weight in the normal range versus the overweight (>120 kg) (major bleeding and all bleeding); however, there was a trend towards significance. There were increased rates of bleeding in both underweight and overweight patients compared to normal weight. Important limitations of this study include a small sample size [27]. Our results show the contrary with an increased bleeding risk in lower weight individuals taking rivaroxaban.

Clinical data on DOAC use in the obese and morbidly obese population is limited. Large phase III trials had analyses of varying weight cutoffs, which make direct comparisons across the heterogeneous groups difficult. Guidelines published in 2016 reported DOACs to be generally safe in obese patients with bleeding rates that are comparable to VKAs. Similar evidence from the AMPLIFY, Hokusai-VTE, EINSTEIN-DVT, EINSTEIN-PE, and ROCKET-AF trials also support the safety of DOACs in patients with a higher BMI [8–12]. The exception to this trend was seen in the AMPLIFY trial, where subgroup analysis showed significantly improved bleeding rates among the obese when comparing apixaban to VKA [8, 23]. Our data supports the safety of DOAC use in patients with higher BMIs by demonstrating no increased bleeding event rates in the apixaban group and lower bleeding event rates in the rivaroxaban group in patients with a BMI over 30.

In addition, our results show that the presence of interacting medications (concomitant use of CYP3A inhibitors and inducers along with antiplatelet agents) is also an independent risk factor for bleeding in patients with BMIs under 25 and under 30. Although our sample size was too small to decipher which medication group was more likely to lead to increased bleeding, we were able to assess that aspirin and plavix alone did not account for these findings. A recent systemic review found no statistical difference for clinical outcomes including major bleeding or intracranial bleeding in patients taking amiodarone and DOAC concomitantly [28]. Both ARISTOTLE and ROCKET-AF post hoc analyses have shown no impact of interacting drugs on bleeding risk although strong CYP3A inhibitors and inducers were excluded. However, ROCKET-AF found increased risk of major bleeding with concomitant dual antiplatelet use in the RE-LY trial and for aspirin in the ROCKET-AF trial [29–31]. Our results suggest that patients taking medication with potential drug interactions should be monitored closely for bleeding.

Importantly, there was no difference in efficacy across different BMIs supporting the findings of larger clinical trials; however, again, sample size in relation to follow-up duration was small, making interpretation difficult. Ideally, a longer follow-up time would allow us to capture more events and more appropriately assess efficacy.

In summary, our results support previous findings that DOACS are safe in the obese; however, the retrospective nature of our study and small sample size remain important limitations. Future efforts to design larger prospective randomized trials inclusive of more patients with BMIs over 25–40 would be required to validate that the safety and efficacy of DOACs are similar across all BMIs.

## Figures and Tables

**Table 1:** Baseline characteristics of study patients.

Characteristics	AF (*N* = 289)	VTE (*N* = 109)	*p* value
Age (yr)	72 ± 12	65 ± 17	<0.0001
Male, no. (%)	165 (57)	46 (42)	0.0079
Race, no. (%)			<0.0001
AA	135 (47)	78 (72)	
White	139 (48)	27 (25)	
Asian	2 (1)	1 (1)	
Hispanic	3 (1)	0	
BMI, no. (%)^*∗*^			0.8337
Underweight (<18.5)	2 (1)	1 (0.9)	
Normal (18.5–24.9)	60 (21)	24 (22)	
Overweight (25.0–29.9)	90 (31)	26 (24)	
Obesity (class I) (30.0–34.9)	63 (22)	28 (26)	
Very obese (class II) (35.0–39.9)	38 (13)	15 (14)	
Extreme obesity (class III) (>40)	36 (13)	13 (12)	
DOAC, no. (%)			
Apixaban	126 (44)	18 (17)	0.2263
2.5 mg twice daily	15 (12)	4 (22)	
5 mg twice daily	111 (88)	14 (78)	
Rivaroxaban	127(44)	87 (79)	0.4532
10 mg once daily	2 (2)	0 (0)	
15 mg once daily	16 (13)	13 (15)	
20 mg once daily	109 (86)	74 (85)	
Dabigatran	36 (13)	3 (3)	0.0828
75 mg twice daily	2 (6)	1 (33)	
150 mg twice daily	34 (94)	2 (67)	
History of hypertension, no. (%)	251 (87)	79 (73)	0.0007
History of diabetes mellitus, no. (%)	75 (26)	27 (25)	0.0090
History of heart failure, no. (%)	98 (34)	16 (15)	0.0002
History of HIV, no. (%)	4 (1)	5(4.6)	0.0553
History of malignancy, no. (%)	41 (14)	13(12)	0.5571
History of lung disease, no. (%)	41 (14)	19 (17)	0.4199
History of treatment failure, no. (%)	12 (4)	10 (9)	0.0506
Presence of any interacting medication, no. (%)	164 (57)	36 (33)	<0.0001
Presence of either aspirin and/or plavix, no. (%)	116 (40)	27 (25)	0.0044
History of prior bleed(s) before AC, no. (%)	21 (7)	11 (10)	0.3553

^*∗*^Categories as described per WHO criteria.

**Table 2:** Safety outcome for NVAF in all patients.

Safety outcome	Rivaroxaban (*N* = 127)		Apixaban (*N* = 126)	
	Event rate (%)	*p* value	Event rate (%)	*p* value
Major bleeding				
BMI <25	0/25 = 0	1.000	4/32 = 12.5	0.036
BMI ≥25	2/102 = 2.0		0/94 = 0	
BMI <30	0/68 = 0	0.214	4/71 = 5.6	0.131
BMI ≥30	2/59 = 3.4		0/55 = 0	

Clinically relevant nonmajor bleeding (CRNMB)				
BMI <25	11/25 = 44.0	0.004	2/32 = 6.3	0.352
BMI ≥25	15/102 = 14.7		13/94 = 13.8	
BMI <30	15/68 = 22.1	0.634	9/71 = 12.7	1.000
BMI ≥30	11/59 = 18.6		6/55 = 10.9	

Combined bleeding^*∗*^				
BMI <25	11/25 = 44.0	0.006	6/32 = 18.8	0.570
BMI ≥25	17/102 = 16.7		13/94 = 24.4	
BMI <30	15/68 = 22.1	0.997	13/71 = 18.3	0.250
BMI ≥30	13/59 = 22.0		6/55 = 10.9	

^*∗*^Combined bleeding is a composite outcome of major bleeding and clinically relevant nonmajor bleeding. Chi-square, and Fisher's exact tests for safety outcomes (major bleeding, CRNMB, and combined bleeding) in patients with BMI <25 versus ≥25 in the NVAF cohort.

**Table 3:** Multivariate analysis of rivaroxaban and CRNMB in overweight patients.

Variables	Odds ratio estimate	95% Wald confidence interval
BMI ≥25 versus <25	5.37	1.50–19.31
Age	1.02	0.98–1.07
Male	0.74	0.23–2.41
Caucasian versus African American	0.54	0.17–1.74
Dose of rivaroxaban	0.96	0.80–1.15
Interacting medications	6.40	1.49–27.57
Aspirin	0.53	0.14–2.04
Plavix	16.68	0.44–630.68
History of prior bleeding	0.20	0.03–1.18

This table presents the multivariate logistical regression model for rivaroxaban and odds ratios for outcome of CRNMB in overweight patients compared to normal weight controls in the NVAF cohort.

**Table 4:** Multivariate analysis of rivaroxaban and CRNMB in obese patients.

Variables	Odds ratio estimate	95% Wald confidence interval
BMI ≥30 versus <30	1.15	0.34–3.89
Age	1.052	1.00–1.11
Male	0.59	0.19–1.84
Caucasian versus African American	0.67	0.22–2.03
Dose of rivaroxaban	0.98	0.82–1.17
Interacting medications	6.73	1.70–26.74
Aspirin	0.63	0.18–2.21
Plavix	9.21	0.25–341.92
History of prior bleeding	0.27	0.05–1.48

This table presents the multivariate logistical regression model for rivaroxaban and odds ratios for outcome of CRNMB in obese patients compared to normal weight controls in the NVAF cohort.

**Table 5:** Safety outcome for VTE in all patients.

Safety outcome	Rivaroxaban (*N* = 86)	
	Event rate (%)	*p* value
Major bleeding		
BMI <25	1/16 = 6.3	1.000
BMI ≥25	4/69 = 5.8	
BMI <30	3/38 = 7.9	0.653
BMI ≥30	2/47 = 4.3	

Clinically relevant nonmajor bleeding (CRNMB)		
BMI <25	2/16 = 12.5	1.000
BMI ≥25	8/69 = 11.6	
BMI <30	4/38 = 10.5	1.000
BMI ≥30	6/47 = 12.8	

Combined bleeding^*∗*^		
BMI <25	3/16 = 18.8	1.000
BMI ≥25	12/69 = 17.4	
BMI <30	7/38 = 18.4	0.866
BMI ≥30	8/48 = 16.7	

^*∗*^Combined bleeding is a composite outcome of major bleeding and clinically relevant nonmajor bleeding. Chi-square and Fisher's exact tests for safety outcomes (major bleeding, CRNMB, and combined bleeding) in patients with BMI <25 versus ≥25 in the VTE cohort.

**Table 6:** Secondary outcome: efficacy and relationship with BMI in all patients.

Efficacy outcome	Rivaroxaban		Apixaban	
	Event rate (%)	*p* value	Event rate (%)	*p* value
Stroke in the AF cohort				
BMI <25	1/25 = 4.0	1.000	0/31 = 0	0.574
BMI ≥25	4/102 = 3.9		3/94 = 3.2	
BMI <30	3/68 = 4.4	1.000	1/70 = 1.4	0.582
BMI ≥30	2/59 = 3.4		2/55 = 3.6	

TIA in the AF group				
BMI 25	0/25 = 0	N/A	0/31 = 0	1.000
BMI ≥25	0/102 = 0		2/94 = 2.1	
BMI <30	0/68 = 0	N/A	2/70 = 2.9	0.503
BMI ≥30	0/59 = 0		0/55 = 0	

Recurrence in the VTE cohort				
BMI <25	2/16 = 12.5	0.315	1/8 = 12.5	1.000
BMI ≥25	4/69 = 5.8		1/11 = 9.1	
BMI <30	3/38 = 7.9	1.000	2/11 = 18.2	0.485
BMI ≥30	3/47 = 6.4		0/8 = 0	

Chi-square and Fisher's exact tests for efficacy outcomes (major bleeding, CRNMB and combined bleeding) in patients with BMI <25 versus ≥25 in both VTE and AF cohorts.

## Data Availability

The datasets used and/or analysed during the current study are available from the corresponding author upon reasonable request.
